# Tunable Optical Diffusers Based on the UV/Ozone-Assisted Self-Wrinkling of Thermal-Cured Polymer Films

**DOI:** 10.3390/s21175820

**Published:** 2021-08-30

**Authors:** Shulan Jiang, Yong Tan, Yong Peng, Jiang Zhao

**Affiliations:** 1 School of Mechanical Engineering and Electronic Information, China University of Geosciences (Wuhan), Wuhan 430074, China; 2 Tribology Research Institute, School of Mechanical Engineering, Southwest Jiaotong University, Chengdu 610031, China; cqyong023@163.com (Y.T.); pengyong@my.swjtu.edu.cn (Y.P.); 3 Hubei Key Laboratory of Ferro & Piezoelectric Materials and Devices, Faculty of Physics & Electronic Sciences, Hubei University, Wuhan 430062, China; zhaojiang@hubu.edu.cn

**Keywords:** PDMS, self-wrinkle, optical diffuser, tunable, strain-dependent

## Abstract

Tunable optical diffusers have attracted attention because of the rapid development of next generation stretchable optoelectronics and optomechanics applications. Flexible wrinkle structures have the potential to change the light path and tune the diffusion capability, which is beneficial to fabricate optical diffusers. The generation of wrinkles usually depends on an external stimulus, thus resulting in complicated fabricating equipment and processes. In this study, a facile and low-cost method is proposed to fabricate wrinkle structures by the self-wrinkling of thermal-cured polymer for tunable optical diffusers. The uncured polydimethylsiloxane (PDMS) precursors were exposed to UV/ozone to obtain hard silica layers and then crosslinked via heating to induce the wrinkle patterns. The wrinkle structures were demonstrated as strain-dependent tunable optical diffusers and the optical diffusion of transmitted light via the deformable wrinkle structures was studied and adjusted. The incident light isotropically diffused through the sample at the initial state. When the wrinkle structures deformed, it showed a more pronounced isotropic optical diffusion with uniaxial tensile strain. The optical diffusion is anisotropical with a further increase in uniaxial tensile strain. The proposed method of fabricating wrinkles by UV/ozone-assisted self-wrinkling of thermal-cured polymer films is simple and cost-effective, and the obtained structures have potential applications in tunable optical diffusers.

## 1. Introduction

Artificial optical diffusers, which are generally made from non-deformable rigid materials, have always been significant parts in many photonics and optoelectronics systems [1,2,3]. For example, liquid crystal displays (LCDs) could effectively reduce the light intensity of LEDs and make the illumination effect more uniform [4,5,6]. Nowadays, optical diffusers are increasing in demand as soft optical components for the development of next generation stretchable optoelectronics and optomechanics applications [7,8,9]. For example, soft optical diffusers can be applied as smart windows [10] or invisible labels [11]. Furthermore, if the specific architectures of the soft diffusers can be easily controlled, the diffusion properties can be dynamically tuned. This interesting optics device is called tunable optical diffuser. Researchers have proposed some potential architectures, such as sinusoidal microwrinkles [12], polystyrene nanospheres/polydimethylsiloxane composite structures [13], hydrogel films [14], UV-cured polymer-dispersed liquid crystals [15] and cylindrical lenslet elastomers [16], to fabricate tunable optical diffusers for dealing with the deformation of photonic devices and controllably tuning the diffusion capability in advanced applications. A common feature of the fabricated diffusers mentioned above is the integration of a flexible elastomer with microstructures to alter the light path.

Surface wrinkles, which are induced by applying a mechanical force beyond the critical value to a system with an elastic substrate supporting a stiff film [17,18], could advisably meet the requirements as tunable optical devices due to the simple preparation process and excellent mechanical properties. Takuya Ohzono et al. [12] proposed a preparation method for a tunable optical diffuser by bonding a thin polymer film onto an elastic substrate and applying a uniaxial compressive strain on the sample to induce controlled sinusoidal wrinkles for changing the light path. However, there is a slip risk for the elastic substrate and microstructures due to the bonding adhesive. Surface modification of the elastomer to form a hard film has the potential to avoid the failure that causes a change of the light path [19,20,21]. In addition, the generation of wrinkles usually depends on an external stimulus, resulting in complicated fabricating equipment and processes. Fortunately, Chandra et al. [22] reported the self-wrinkling of UV-cured polymer films without any external stimulus on the elastomer. However, the chemical composition of the UV-cured polymer is complex, so the materials are not readily available. To our knowledge, UV/ozone-assisted thermal-cured self-wrinkling micropatterns for the application of tunable optical diffusers have not been studied, which is promising for stretchable optoelectronics. In this regard, ubiquitous PDMS is an ideal flexible optical device substrate because of its exceptional light transparency, stretchability and oxidizability [23,24].

Here, we have proposed a simple and low-cost method for the fabrication of tunable optical diffusers based on UV/ozone-assisted self-wrinkling thermal-cured polymer films. The self-wrinkle structures with good mechanical stretchability were fabricated on UV/ozone-treated PDMS precursor surfaces before curing. We have investigated the influences of substrate roughness, ratio of base and crosslinking agent, and UV/ozone oxidation time to the wavelengths and amplitudes of the self-wrinkle structures. The diffusion properties were measured using a red laser beam. The area and shape of the diffusion spot could be reversibly tuned by a different uniaxial strain.

## 2. Materials and Methods

The PDMS (Sylgard 184, Dow and Corning, Midland, MI, USA) precursor was prepared by mixing a base and crosslinking agent at ratios of 8:1, 10:1 and 12:1, respectively. The base is a poly-(dimethyl-methylvinylsiloxane) prepolymer with a small amount of platinum catalyst and the curing agent is a mixture of vinyl-endcapped PDMS precursors and poly-(dimethyl-methylhydrogenosiloxane) precursors as cross-linkers. After degassing in a vacuum kettle, the precursor mixtures were spin-coated onto the glass substrate (12 × 12 × 1 mm^3^) for 60 s at 500, 1000 and 3000 rpm, respectively. Then, the samples remained standing for 15 min to avoid the flow of the PDMS precursor on the substrate. The uncured PDMS samples were then exposed to a 184.9 nm and 253.7 nm UV source (PSDP UV-8T, Novascan, Ames, IA, USA) for 2, 6 and 10 min, respectively. Next, the treated samples were cured by heating them on a hotplate for 60 min with various curing temperatures of 80, 90 and 100 °C. The self-wrinkle structures were fabricated by thermal polymerization and cross-linking of UV/ozone-treated uncured PDMS samples, and were then peeled off the PDMS samples.

The morphologies of the self-wrinkle surfaces were observed by an atomic force microscope (AFM) and a three-dimensional (3D) optical surface profiler (SuperView W1). The wavelengths (Λ) of the self-wrinkle structures were acquired by locating the line profile on the image, and the amplitudes (A) were approximately described by the surface roughness (Ra).

The optical diffusion properties based on the flexible samples were tested by an ordinary laser diode (wavelength: λ = 650 nm) with elongated samples. The self-wrinkle surface was deformed by the balancing weights to provide the uniaxial stretching strain. The transmitted laser beam was monitored on a paper screen placed at 100 cm from the samples. The visible laser spot on the paper screen was detected using a digital camera (70D, Canon) and the distance between the camera and the screen was 50 cm. The spot area was determined by optical images after converting them to 8-bit and auto-thresholding using ImageJ software.

## 3. Results and Discussion

**Figure 1:**
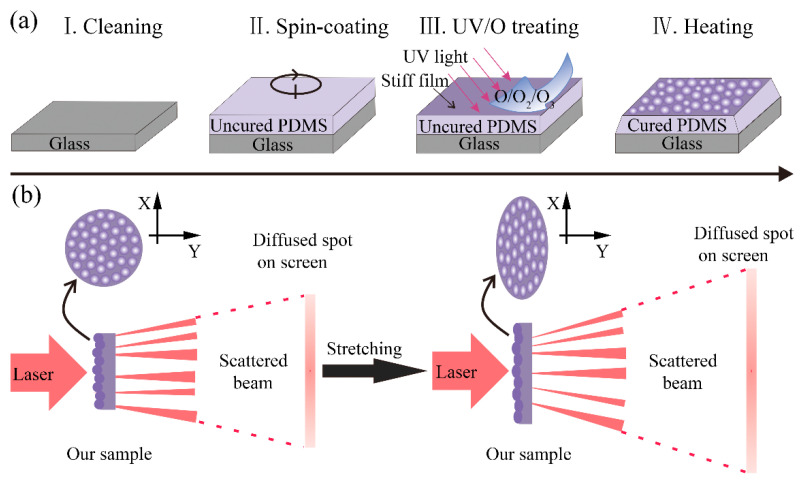
(**a**) Mechanism of self-wrinkling in thermal-cured polymer films. (**b**) Working principle of the optical diffuser based on the stretchability of the self-wrinkle structures.

Before curing, the base and curing agent of the PDMS precursors were mixed with different ratios. When the uncured PDMS was exposed to an ultra-violet light and oxidized by the UV/ozone system which could continuously generate atomic oxygen and ozone, a thin and stiff SiO_x_ layer emerged due to the photo-oxidation reaction [25]. During conventional curing on a hotplate, the PDMS would shrink because of the cross-linking of the monomer and a decrease in the total volume [26,27]. The temperature difference between the upper and lower surface of PDMS on the hotplate would also cause shrinkage. Hence, this work utilized the interface mismatch between the bottom cured PDMS and the top stiff SiO_x_ layer to generate surface self-wrinkle patterns autonomously (Figure 1a). This is different from the reported external-stimulus-induced formation of wrinkle patterns [28,29]. The intermolecular force that is formed during PDMS curing is an internal stress which contributes to the interface mismatch between the elastomer and the rigid film. The AFM surface profile in Supplementary Materials  Figure S1a shows the typical morphology of the self-wrinkle, which appeared like a microlens array. Figure 1b illustrates the working principle of the tunable optical diffuser. For the original state of the diffuser, the laser transmitted the self-wrinkle sample, and the beam was refracted to achieve divergence. The size and shape of the diffused spot of the optical diffuser can be adjusted by the deformation of the self-wrinkle structures, which was triggered by the simple uniaxial tensile strain. In this study, the tensile strain was applied to the flexible optical diffuser for deforming the self-wrinkle to alter the direction of light path propagation. It is worth mentioning that the degree of diffusion was continuously and reversibly tunable.

The spin-coating speed, ratio of base and crosslinking agent, UV/ozone oxidation time and PDMS curing temperature will affect the formation of the self-wrinkle structures. To better understand the influence of preparation parameters to the self-wrinkle patterns, orthogonal experiments were designed to measure the self-wrinkle average wavelengths (Λ) and amplitudes (A) along with the spin-coating speed, ratio of base and crosslinking agent, UV/ozone oxidation time and PDMS curing temperature (Table 1). Particularly, experiment number zero was used as a comparison, which is a bare PDMS without self-wrinkle structures. As shown in Figure S1b, the Ra of 3.04 nm was measured on the bare PDMS surface which was not treated by the UV/ozone system before PDMS curing. Furthermore, the experiments with smooth glass and frosted glass as substrates were carried out to explore the influence of the substrate surface roughness for the self-wrinkle structures. The optical micrographs in Figure S1c,d, show the morphology of smooth and frosted glass substrates, respectively.

**Figure 2:**
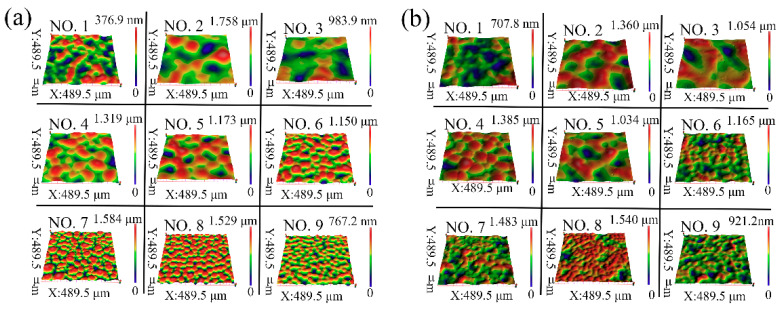
3D optical images of self-wrinkle structures on (**a**) smooth glass substrates and (**b**) frosted glass substrates, respectively.

Figure 2a,b show the 3D images of the samples fabricated on smooth and frosted glass substrates, respectively, with experiment number 1–9 at Table 1. It demonstrates that the frosted and smooth substrates have little effect on the morphologies of the self-wrinkle structures.

**Figure 3:**
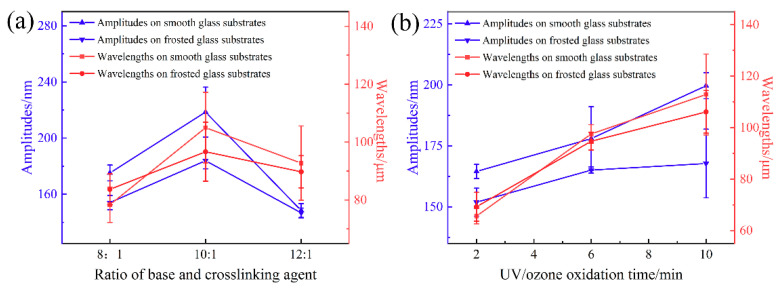
The relationships of wavelengths and amplitudes along with the (**a**) ratio of base and crosslinking agent and (**b**) UV/ozone oxidation time on the smooth glass substrates and frosted glass substrates, respectively.

The formation of self-wrinkle structures is affected by the spin-coating speed, ratio of base and crosslinking agent, UV/ozone oxidation time and curing temperature. The changes in structure wavelengths and amplitudes on the smooth and frosted glass substrates that are affected by the above four factors were studied, and the results are shown in Figure 3 and Figure S2. Similar tendencies are observed for the wavelengths and amplitudes on the two substrates. The wavelengths of the self-wrinkle on the two substrates are slightly different, and the amplitude on the smooth glass substrate is larger. Figure 3 shows the relationships between the A and the Λ with the PDMS ratio of 8:1, 10:1 and 12:1. The ratio of 10:1 produces the largest Λ of 104.99 μm and an A of 218.57 nm on the smooth glass substrates, and the largest Λ of 96.67 μm and an A of 183.97 nm on the frosted glass substrates. In Figure 3b, the Λ and A gradually increase with the oxidation time. The Λ increases from 65.64 to 112.87 μm and from 69.26 to 106.1 μm, and the A increases from 164.5 to 199.67 nm and from 151.9 to 167.77 nm for an increase in oxidation time from 2 to 10 min on the smooth glass substrates and frosted glass substrates, respectively. These results indicate that the longer the oxidation time, the thicker the SiO_x_ film. This agrees with prior reports in the literature [30], such that, as the thickness of the rigid film increases, the Λ and A of the self-wrinkle structures increase accordingly. As shown in Supplementary Materials Figure S2a, the A decreases and the Λ increases with increasing the PDMS spin-coating speed (i.e., the PDMS thickness decreases with increasing PDMS spin-coating speed). As the spin-coating speed is increased from 500 to 3000 rpm, the Λ of the self-wrinkle structures monotonically decreases from 128.07 to 50.08 μm, and the A increases from 156.93 to 201.77 nm on the smooth glass substrates. Similarly, the Λ decreases from 111.93 to 62.52 μm, and the A increases from 155.92 to 169.88 nm on the frosted glass substrates. The relationships of Λ and A with PDMS curing temperature are shown in Supplementary Materials Figure S2b. The Λ increases slightly from 85.96 to 102.83 μm and from 87.19 to 98.27 μm with the increase in the curing temperature from 80 to 100 °C on the smooth glass substrates and frosted glass substrates, respectively. For the curing temperature of 90 °C, the largest A values are 241.27 nm and 191.31 nm, respectively.

**Figure 4:**
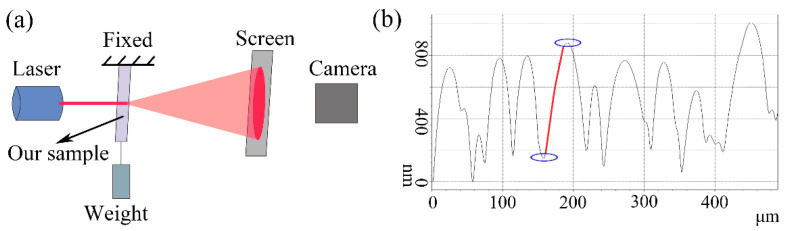
(**a**) Experimental setup for the observation of the diffused laser spot. (**b**) Cross-sectional profile of the self-wrinkle.

We have explored the application of the microlens self-wrinkle structures on the tunable optical diffusers and have tested the transmission diffusion property under different uniaxial tensile strains. The experimental setup to measure the transmission diffusion property is shown in Figure 4a. The transmission path of light was analyzed through geometric optics. The wavelength (λ) of the red incident laser is 650 nm, which is much smaller than the typical wavelength (Λ ≈ 50~142 μm) of the self-wrinkle structures, and the effects of interference and diffraction are ignored here [12]. The laser travels through the sample in a straight line and the laser refracts at the interface of air, PDMS and SiO_x_ film. In this study, for simplicity, the refractive indexes n and n_s_ of the PDMS and SiO_x_ film are regarded as constant when the diffuser is deformed [12].

**Figure 5:**
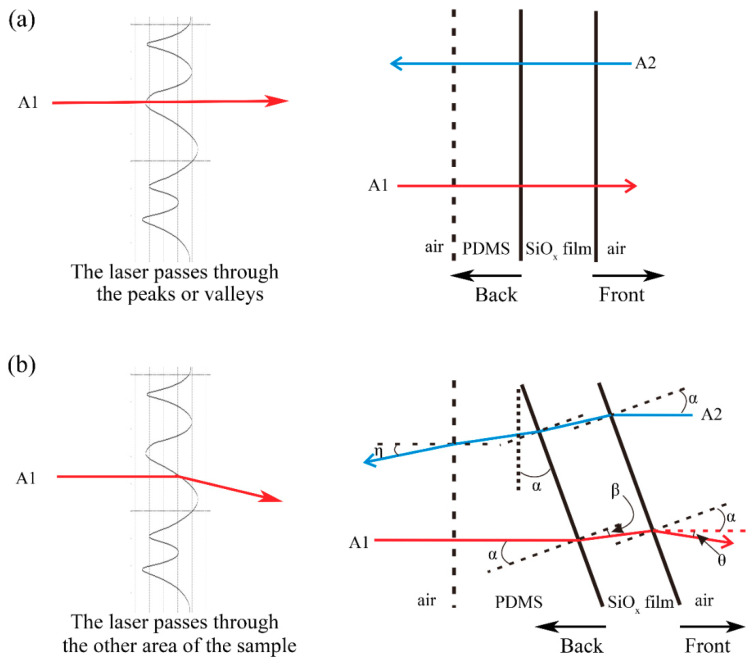
Schematic of the geometry for lasers passing through the (**a**) peaks or troughs and (**b**) the other positions of the self-wrinkle.

The cross-sectional profile of the microlens self-wrinkle structures could be approximately fitted to a sinusoidal structure (Figure 4b). We have also demonstrated the similar cross-sectional profiles of different areas of the self-wrinkle structure sample in Supplementary Materials Figure S3. When the laser passes through a certain position of the sinusoidal structure, the curvature radius of the SiO_x_ film tends toward infinity at this position in geometric optics. Figure 5a shows that the laser direction does not change when the laser passes through the peaks or valleys of the sinusoidal structure. Here, the interface between the SiO_x_ film and air is defined as the front, and the interface between the PDMS layer and SiO_x_ film is defined as the back. When the laser passes through another area of the sample, the laser direction will be refracted. As shown in Figure 5b, a horizontal light, A1, irradiates the diffuser from the back side. The angle (*α*) is defined as the angle between the horizontal incident laser and the normal position. The angle between the vertical direction and the back SiO_x_ film is also equal to *α*. The first refraction of the laser occurs at the PDMS-SiO_x_ film interface and refracts again at the SiO_x_ film-air interface. When the light transmits from the front side, the incident laser, A2, is refracted three times. *β* is the angle defined in Figure 5b. The final deflection angle (*θ*) between the second emitted light and the horizontal direction determined the diffusion ability of the optical diffuser. According to Snell’s Law, the refraction angle could be calculated as $n\sin\alpha =n_{s}\sin\beta$, $n\sin\alpha =n_{s}\sin\beta$ and $n_{s}\sin\beta =n_{0}\sin\left(\alpha +\theta \right)$, for the first and second refractions, respectively, where *n*_0_ = 1 is the refractive index of air. As a result, sin(α+θ)=nsinα, where the deflection angle *θ* is independent of *n_s_*.

**Figure 6:**
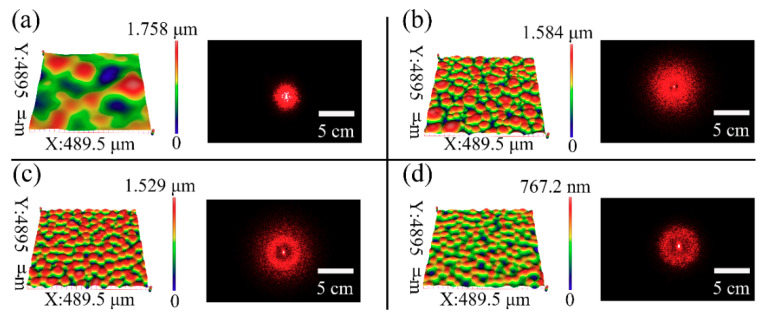
(**a**–**d**) 3D optical images of self-wrinkle structures and the corresponding diffused spots for the samples fabricated with the parameters of experiments 2, 7, 8 and 9 in Table 1, respectively.

The diffusion ability of the typical samples 2, 7, 8, and 9 were evaluated, as shown in Figure 6. The aspect ratios (R = A/Λ) of the microlens self-wrinkles structures for samples 2, 7, 8 and 9 are 1.78 × 10^−3^, 5.06 × 10^−3^, 4.59 × 10^−3^, and 1.95 × 10^−3^, respectively. The visual shift of the diffused spot is very clear, which can be observed by the naked eye. The diffused spot on the screen becomes noticeably large with the increase in the self-wrinkle aspect ratio. This phenomenon agrees with prior reports in the literature [12,14], which showed that a larger aspect ratio (R) resulted in broad diffused spots. In addition, the light intensity is greater at the center of the diffuse spot and a hollow ring appears in the center of the diffuse spot in Figure 6b–d, which are caused by the peaks or valleys of the sinusoidal structure (shown in Figure 5a) and the excessive deflection angle θ (shown in Figure 5b), respectively.

**Figure 7:**
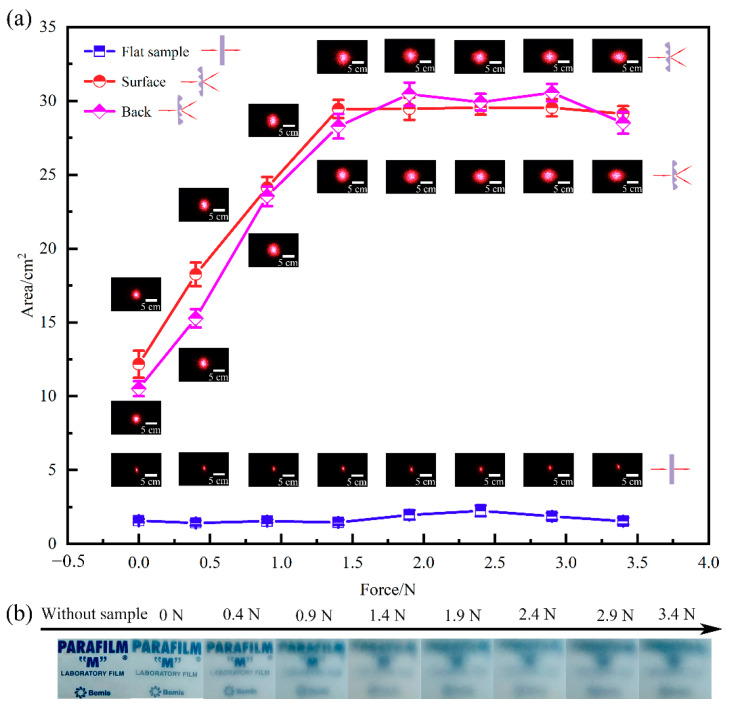
(**a**) The relationships between the spot area and applied force when the laser irradiates a flat sample, and wrinkle structures samples from the front side and back side, respectively. (**b**) Transmitted images of the PARAFILM logo through the sample with different forces.

In this work, the typical sample fabricated with the ratio of 10:1, spin-coating speed of 500 rpm, UV/ozone oxidation time of 6 min and curing temperature of 90 °C was utilized to explore the tunability of optical diffusion. The optical images of the strain-dependent diffusion of the transmitted laser spots on the screen are shown in Figure 7a. It shows that the area of laser spots remain by ~0.5% when the laser passes through the bare PDMS samples even with the increase in strain. When the laser passes through the self-wrinkle sample from the back side, the laser is diffused into a large circle spot without strain, and the laser spot area (~3%) is about six times that of the bare PDMS sample. When the laser passes through the self-wrinkle sample from the front side, the laser spot area is close to the area that transmits from the back side. It shows that the diffusion effect is mainly caused by the self-wrinkle structures and the thin SiO_x_ layer, and there are similar irradiation results from the back or front side. As shown in Figure 5b, horizontal lasers A1 and A2 irradiate the sample from the back side and the front side, respectively. According to the experimental results, it could infer the laser beam deflection angle *θ* = *η*.

As shown in Figure 7a, the laser spot gradually increases with the uniaxial tensile strain for the self-wrinkle sample. The diffusion spot area increases when the applied force increases from 0 N to 1.4 N (including plate weight). The strain changes the morphology of the self-wrinkle structures, so the aspect ratio changes accordingly. The incident angle (*α*) in geometric optics increases isotropically with the force. It may be the main diffusion reason of the strain-dependent optical diffuser. It shows that the shape of the diffusion spot gradually turns to elliptical with a force over 1.4 N. At this stage, the microlens arrays gradually turn into ellipsoids due to the uniaxial stretching strain, thus resulting in the transformation of a circular spot to an elliptical. Additionally, the diffusion spot area is kept constant and is a strain-dependent anisotropic diffusion. It shows similar results when the laser irradiates the diffuser from the front and the back of the sample, as shown in Figure 6a. The diffuser is probably independent of the direction of the laser incidence. The above results show that the optical diffusion is reversibly tuned by adjusting the magnitude of the applied force.

We also tested the transmittance of the diffuser by observing the PARAFILM logo through the self-wrinkle sample with different forces, and the results are shown in Figure 7b. The logo is ultraclear without a diffuser. When the uniaxial tensile force is increased from 0 to 1.4 N, the logo images gradually become blurry, and the transmittance turns bad. When the tensile force surpasses 1.4 N, the transmittance remains unchanged. The first-stage isotropic diffusion can significantly affect the light transmittance, and the second-stage isotropic diffusion doesn’t change the light transmittance.

**Figure 8:**
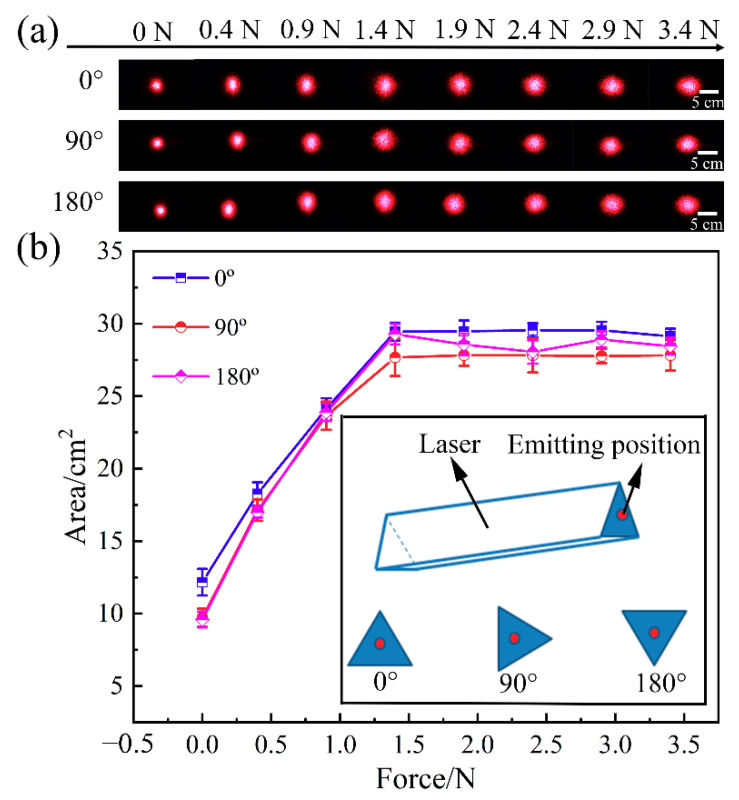
(**a**) Laser spots and (**b**) diffused area of the microlens self-wrinkle sample tested with different forces when laser irradiates the diffuser with rotating the laser angle of 0°, 90° and 180°, respectively.

We repeated the above experiments of the frontal incidence laser by rotating the laser at different angles to eliminate the effects of the laser light source. As shown in Figure 8, when the laser irradiated the diffuser at the angles of 0°, 90°and 180°, the shape and area of the spots had similar tendencies with the increase in strain. Therefore, the spot shape of the laser will not affect the diffusion performance of the tunable optical diffuser based on the self-wrinkling microstructure. In our experiment, the same sample of the optical diffuser was tested repeatedly and showed good durability.

## 4. Conclusions

In summary, tunable optical diffusers fabricated by the UV/ozone-assisted self-wrinkling of thermal-cured polymer films have been studied. Taking advantage of the oxidation, the system of a stiff SiO_x_ layer on top closely fitting the underlying PDMS precursor was prepared, and microlens self-wrinkle patterns were fabricated by curing PDMS without an external stimulus. The microlens self-wrinkle patterns provide conditions for the fabrication of a tunable optical diffuser. This work indicates the strain-dependent tunable diffusion of a red laser beam through the diffuser from the front or back side. The diffusion is isotropic, and the spot area increases linearly from 0 to 1.4 N of uniaxial tensile force. When the force is over 1.4 N, the diffusion is anisotropic, and the spot area remains constant. This work demonstrates that the present microlens self-wrinkle structures could be used as a mechanically tunable optical diffuser by tuning the applied force. We also believe that the diffusion formed by the microlens self-wrinkle structures would find unique applications.

## Figures and Tables

**Table 1:** Orthogonal experiments to explore the preparation parameters of self-wrinkle structures.

Experiment Number	Spin-Coating Speed/rpm	Ratio	Oxidation Time/Min	Curing Temperature / °C
0	500	10:1	0	90
1	500	8:1	2	80
2	500	10:1	6	90
3	500	12:1	10	100
4	1000	8:1	6	100
5	1000	10:1	10	80
6	1000	12:1	2	90
7	3000	8:1	10	90
8	3000	10:1	2	100
9	3000	12:1	6	80

## Data Availability

Not applicable.

## Supplementary Materials

The following are available online at https://www.mdpi.com/article/10.3390/s21175820/s1, Figure S1: AFM images of (a) the typical self-wrinkle structures and (b) the bare PDMS sample without self-wrinkle structures. The optical micrographs of (c) smooth glass substrate and (d) frosted glass substrate. Figure S2: The relationships of wavelengths and amplitudes along with the (a) spin-coating speed and (b) PDMS curing temperature on the smooth glass substrates and frosted glass substrates, respectively. Figure S3: Cross-sectional profiles of different areas of the self-wrinkle structure sample.
