# Supplementary material for: Tunable Optical Diffusers Based on the UV/Ozone-Assisted Self-Wrinkling of Thermal-Cured Polymer Films
Source: Sensors (Basel). 2021 Aug 30;21(17):5820. doi: 10.3390/s21175820 (PMC8434521; doi:10.3390/s21175820)
Supplement: Supplementary file 1 [file sensors-21-05820-s001.zip › sensors-1318069-supplementary.pdf]

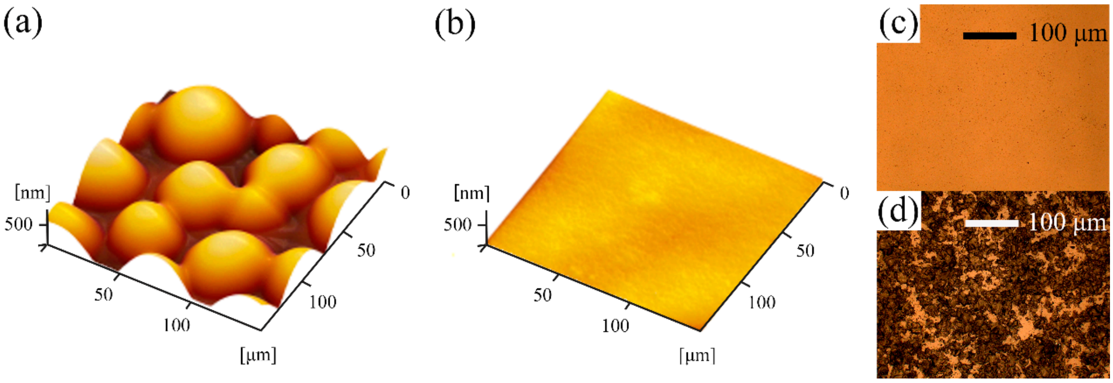

Figure S1. AFM images of (a) the typical self-wrinkle structures and (b) the bare PDMS sample without self-wrinkle structures. The optical micrographs of (c) smooth glass substrate and (d) frosted glass substrate.

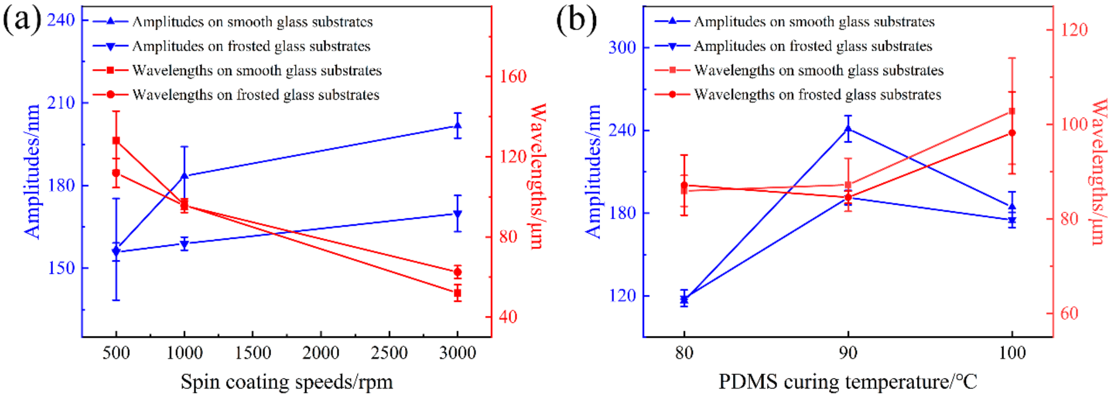

Figure S2. The relationships of wavelengths and amplitudes along with the (a) spin-coating speed and (b) PDMS curing temperature on the smooth glass substrates and frosted glass substrates, respectively.

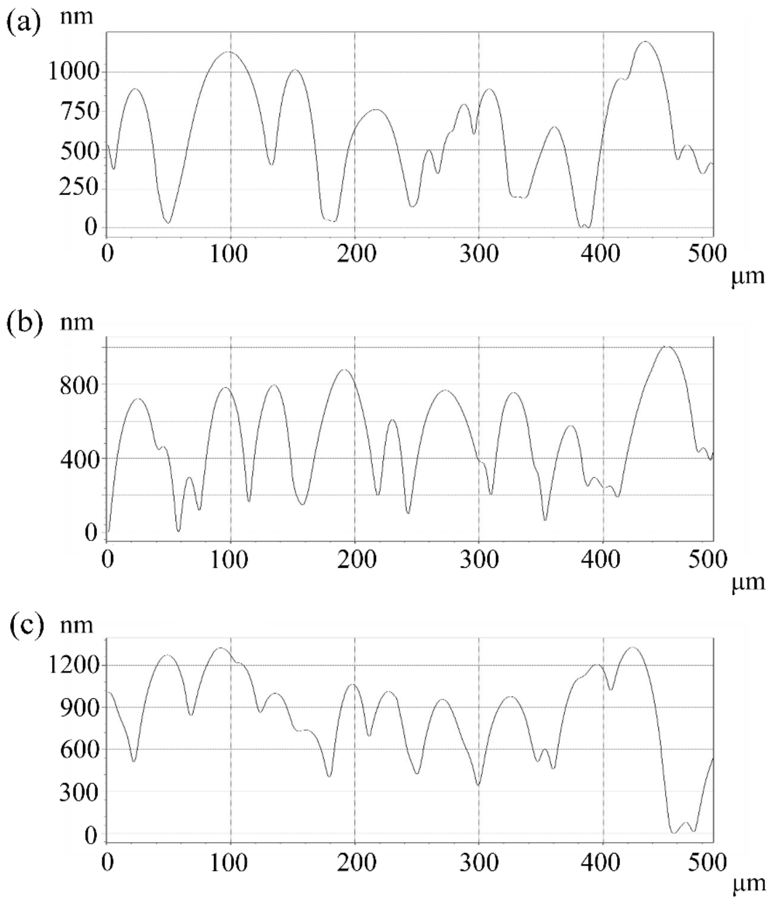

Figure S3. Cross-sectional profiles of different areas of the self-wrinkle structure sample.
